# Transformational Leadership, Transactional Contingent Reward, and Organizational Identification: The Mediating Effect of Perceived Innovation and Goal Culture Orientations

**DOI:** 10.3389/fpsyg.2017.01754

**Published:** 2017-10-18

**Authors:** Athena Xenikou

**Affiliations:** Department of Aeronautical Sciences, Hellenic Air Force Academy, Athens, Greece

**Keywords:** organizational culture, transformational leadership, transactional contigent reward, cognitive identification, affective identification, self-concept, organizational identification

## Abstract

**Purpose:** The aim of this research was to investigate the effect of transformational leadership and transactional contingent reward as complementary, but distinct, forms of leadership on facets of organizational identification via the perception of innovation and goal organizational values.

**Design/Methodology/Approach:** Three studies were carried out implementing either a measurement of mediation or experimental-causal-chain design to test for the hypothesized effects.

**Findings:** The measurement of mediation study showed that transformational leadership had a positive direct and indirect effect, via innovation value orientation, on cognitive identification, whereas transactional contingent reward was more strongly related to affective, rather than cognitive, identification, and goal orientation was a mediator of their link. The findings of the two experimental-causal-chain studies further supported the hypothesized effects. Transformational leadership was found to lead subordinates to perceive the culture as more innovative compared to transactional contingent reward, whereas transactional contingent reward led employees to perceive the culture as more goal, than innovation, oriented. Finally, innovation, compared to goal, value orientation increased cognitive identification, while goal orientation facilitated affective, rather than cognitive, identification.

**Implications:** The practical implications involve the development of strategies organizations can apply, such as leadership training programs, to strengthen their ties with their employees, which, in turn, may have a positive impact on in-role, as well as extra-role, behaviors.

**Originality/Value:** The originality of this research concerns the identification of distinct mechanisms explaining the effect of transformational leadership and transactional contingent reward on cognitive and affective identification applying an organizational culture perspective and a combination of measurement and causal mediation designs.

## Introduction

Individuals tend to define themselves, at least partly, in terms of the organization(s) in which they are members. The perception of oneness with or belongingness to an organization is the essence of organizational identification, which reflects the extent to which group membership is incorporated in the self-concept (Ashforth and Mael, 1989; Ashforth, 2016). There is ample empirical evidence showing that organizational identification is positively associated with numerous favorable work outcomes such as performance of extra-role behaviors, increased job satisfaction, voice-behavior (i.e., making constructive criticism and suggestions for change), innovative behavior, organizational citizenship behaviors, and lower turnover intentions (Van Knippenberg and Van Schie, 2000; Van Dick et al., 2004; Riketta, 2005; Olkkonen and Lipponen, 2006; Van Knippenberg and Sleebos, 2006; Lipponen et al., 2008; Randsley de Moura et al., 2009; Liu et al., 2010; Johnson et al., 2012; Lee et al., 2015).

Organizational identification was initially conceptualized as the cognitive awareness that the self is part of the organization, not necessarily related to any affective states (Ashforth and Mael, 1989; Mael and Ashforth, 1992; Dutton et al., 1994). More recently, however, researchers have put emphasis on both the cognitive and affective facets of organizational identification (Ellemers et al., 2004; Kreiner and Ashforth, 2004; Johnson et al., 2012). The cognitive component concerns awareness of membership in the social group organization, and the affective component refers to an individual member's feelings in relation to a particular organization.

The main purpose of this research was to investigate the joint effect of leadership behavior patterns and culture value orientations on the two different facets of organizational identification. On the basis of an important theoretical account of organizational identification that involves the self-engaging processes (i.e., making organizational members' collective identity become salient) of transformational and charismatic leadership (Bass, 1985; Shamir et al., 1993; Lord et al., 1999; Bass et al., 2003) the effect of transformational leadership and transactional contingent reward on the two facets of organizational identification (cognitive/affective) was examined. To this end, in the present research transformational leadership and transactional contingent reward were examined because, as extensively discussed in the next section, they constitute the defining and consistent behavioral patterns that lead to positive employee attitudes, such as affective organizational commitment, and augmented performance. Moreover, it was investigated whether leadership behavior influences the perceptions employees hold of their organization's culture by focusing on two culture dimensions that have been theoretically and empirically shown to constitute core aspects of organizational cultures (Quinn, 1988; O'Reilly et al., 1991; Hartnell et al., 2011, 2016; Xenikou and Furnham, 2013), namely, innovation and goal orientations, and were expected to work as distinctive mechanisms differentiating the effects of transformational leadership and transactional contingent reward on organizational identification.

### The relative validity of leadership styles in predicting cognitive/affective identification

In the past 30 years or so transformational leadership theory (Bass, 1985) has stimulated an intense empirical investigation of how transformational and transactional leadership behaviors are related to various important work outcomes, such as organizational commitment and identification (Bycio et al., 1995; Dvir et al., 2002; Avolio et al., 2004; Walumbwa et al., 2004; Simosi and Xenikou, 2010; Effelsberg et al., 2014), and work performance (Piccolo and Colquitt, 2006; Walumbwa et al., 2008; Wang et al., 2011; Carter et al., 2013). Bass has put forward the idea that the terms of transformational and transactional leadership, first introduced by Burns (1978), can be of great importance in our attempt to understand leadership in organizations.

Departing, however, from Burns' ideas, Bass and his colleagues (Bass, 1985, 1999; Hater and Bass, 1988; Waldman et al., 1990; Yammarino et al., 1993; Avolio et al., 1999; Bass and Riggio, 2006) have argued that leaders typically exhibit a variety of patterns of transformational and transactional leadership; most leaders do both but in different amounts. According to this model (see Avolio et al., 1999), transformational leadership consists of three core dimensions, that is, charisma/inspirational, intellectual stimulation, and individualized consideration. Through charisma transformational leaders engender respect and inspiration to their followers, through intellectual stimulation leaders encourage creativity and divergent thinking, and finally, through individualized consideration leaders are supportive to employees' needs and aspirations. Moreover, the model incorporates transactional contingent reward that constitutes the most effective component of transactional leadership (as compared to management-by-exception which has a punitive character) in terms of facilitating positive work attitudes and high performance. Transactional contingent reward refers to how the leader clarifies the role and task requirements for subordinates as well as the performance criteria and the rewards upon accomplishing desired goals (Bass, 1985).

Overall, transformational and transactional leadership are considered to be complementary forms of leadership that are both conducive to organizational effectiveness (Bass, 1985; Waldman et al., 1990; Avolio et al., 1999). While transactional leadership qualities can be satisfying and effective, transformational leadership behavior has been shown to add substantially to the impact of transactional leadership on effectiveness as well as followers' satisfaction (augmentation hypothesis; Hater and Bass, 1988; Waldman et al., 1990; Bycio et al., 1995). This augmentation effect refers to the extent to which transformational leadership builds on the transactional base in contributing to more positive work attitudes, extra effort and higher employee performance. In addition, more recent meta-analytic results have shown that transformational leadership and transactional contingent reward have comparable levels of validity (Lowe et al., 1996; Judge and Piccolo, 2004; Wang et al., 2011) supporting the idea that transformational leadership is not a substitute for transactional leadership behaviors toward achieving higher levels of effectiveness and positive employee attitudes.

In a number of studies, however, there were moderate to high correlations between the elements of transformational leadership and transactional contingent reward, which is the most effective component of transactional leadership in terms of a variety of organizational outcomes (Bycio et al., 1995; Avolio et al., 1999; Tejeda et al., 2001; Judge and Piccolo, 2004). These findings support the notion that transformational leadership and transactional contingent reward may indeed be tapping complementary mechanisms in the leadership process (the augmentation hypothesis), but they also posit the question of their relative validity. The main aim of this research was to examine whether transformational leadership and transactional contingent reward are complementary, but distinct, forms of leadership that have an impact on followers' identification via different mechanisms.

More specifically, transformational leaders focus on changing outdated or dysfunctional elements of the organization by stimulating creativity and innovation among followers. They provide their subordinates with intellectual stimulation concerning new ways to think about problems or to do things, and encourage them to participate into problem identification and idea generation (Bass, 1985; Howell and Avolio, 1993; Avolio et al., 1999; Bass et al., 2003). The cognitive component of organizational identification was expected to be more strongly influenced by transformational leadership behaviors compared to transactional contingent reward on the grounds of cognitive flexibility promoted by transformational leaders. Transformational leaders reframe the situation and provide creative insight prompting higher levels of creativity among their subordinates (Sosik et al., 1997; Shin and Zhou, 2003, 2007; Gong et al., 2009; Henker et al., 2015). The sense of innovative direction the transformational leader provides to subordinates facilitates flexibility in information processing, breaking out of perceptual and cognitive frames, and using broad and inclusive social categories, and, therefore, enhances cognitive identification with the organization (i.e., perceptions of similarity among organizational members). As far as affective identification is concerned, transformational leaders are able to inspire organizational members by providing meaning and challenge to their work, and involve them emotionally with the vision they communicate to the group. There are consistent research findings showing that transformational and charismatic leadership are both positively associated with affective identification and commitment (Bycio et al., 1995; Avolio et al., 2004; Walumbwa et al., 2004; Xenikou, 2014). It was, therefore, hypothesized that transformational leadership behaviors are positively related to cognitive and affective identification.

On the other hand, transactional contingent reward operates at a more explicit, contract-based, level by clearly specifying role and task requirements for subordinates, setting performance criteria, and providing rewards for effort expenditure, as well as goal achievement. Employees are motivated to put effort toward doing their job well to achieve a variety of positive outcomes and rewards (Bass, 1985; Avolio et al., 1999; Goodwin et al., 2001; Bass et al., 2003; Wang et al., 2011). Striving to accomplish goals (positive activation) is related to the experience of positive affect as, for example, when a person feels active, enthusiastic, excited, proud, and strong (Watson et al., 1988). Positive activation reflects the nature of transactional contingent reward in comparison to management-by-exception since the latter has the punitive character of the transactional leadership construct (Avolio et al., 1999; Bass et al., 2003). On similar lines, affective identification is thought to be associated with positive feelings about organizational membership, and affective commitment has been shown to be associated with the experience of more positive affective states at work (Albert et al., 1998; Herrbach, 2006). It was, therefore, anticipated that transactional contingent reward is positively related to affective identification beyond the effect of transformational leadership behaviors.

**Figure 1:**
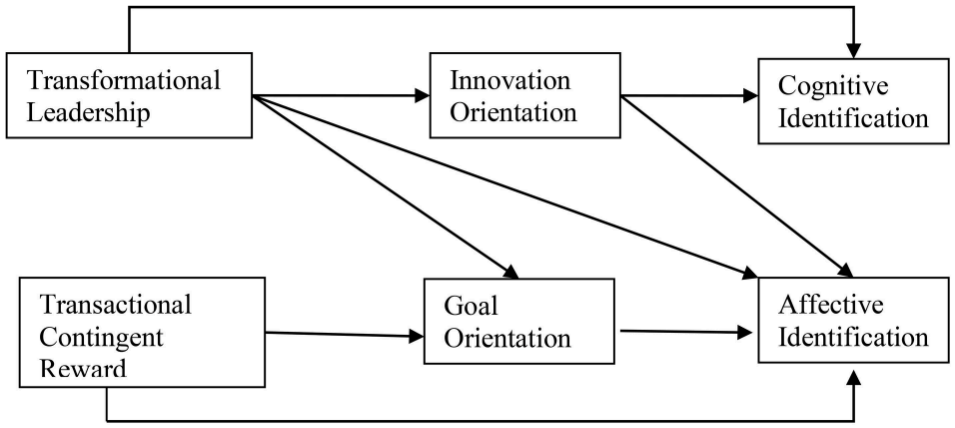
Theoretical model of the link between transformational leadership, transactional contingent reward, and organizational identification as mediated by goal and innovation culture orientations.

On the basis of the aforementioned theoretical analysis and the existing empirical findings, the following hypotheses were formulated (see Figure 1):

**Hypothesis 1**. Transformational leadership was expected to be positively related to cognitive and affective identification.

**Hypothesis 2**. Transactional contingent reward was expected to be positively related to affective identification.

### The mediating effect of culture orientations in the relation between leadership and identification

Organizational cultures are contextual factors that have a profound influence on the emergence and effectiveness of leadership in organizational settings, especially as organizations grow and become differentiated by various divisions and departments. On the other hand, an essential aspect of leadership in organizations is to influence the values, beliefs, and behavioral expectations that organizational members hold, and therefore, leaders put a lot of effort into the maintenance, development, and change of organizational cultures (Bass and Avolio, 1993; Trice and Beyer, 1993; Waldman and Yammarino, 1999; Xenikou and Simosi, 2006; Berson et al., 2008; Schein, 2010; Hartnell and Walumbwa, 2011). Since organizational identification refers to the process of internalizing the organization by viewing its central and more or less enduring qualities as self-defining (Ashforth, 2016), it is arguably the individual's subjective perception of the work context that is more important in predicting individual employee identification with the organization. Therefore, this research examines the effect of leadership style on employees' organizational identification via its impact on perceptions of organizational value orientations.

More recently, there is accumulated empirical evidence showing culture orientations to mediate the impact of leadership styles on important organizational outcomes, such as performance, innovation, and employees' commitment (Ogbonna and Harris, 2000; Lok et al., 2005; Xenikou and Simosi, 2006; Sarros et al., 2008; Simosi and Xenikou, 2010). Regarding employees' commitment, Lok et al. found support for the mediating role of innovative and supportive subcultures in the relation between consideration leadership and commitment in a sample of nurses working at hospital settings. Ogbonna and Harris demonstrated that supportive and participative leadership were associated with performance via innovative and competitive cultures. Similarly, Xenikou and Simosi showed that transformational leadership and humanistic culture orientation had an indirect effect on business unit performance via achievement culture orientation.

This research draws on the competing values model (Quinn, 1988) to examine the impact of transformational leadership and transactional contingent reward on cognitive and affective identification. Transformational leadership behaviors are often targeted at changing outdated elements of an organization's culture, whereas transactional contingent reward behaviors work within culture as it exists (Bass and Avolio, 1993). An innovation value orientation has been shown to be associated with enhanced organizational commitment (McKinnon et al., 2003; Lok et al., 2005; Taylor et al., 2008), and is hypothesized to play a mediating role in the relation between transformational leadership behaviors and organizational identification. An organizational innovation orientation is reinforced by transformational leadership behaviors, and enhances cognitive flexibility making perceptions of similarity among individual employees become salient. Moreover, the promotion of innovation values and creativity within an organizational setting facilitates the psychological merging of self and organization leading employees to feel positively about their organizational membership. Therefore, the following hypothesis was formulated (see Figure 1):

**Hypothesis 3**. Innovation value orientation was expected to serve as a mediator between transformational leadership and both facets of identification.

Another key element of cultural manifestation is a goal value orientation that promotes individual achievement, performance indicators, accountability, and contingent reward (Quinn, 1988; van Muijen et al., 1999; Hartnell et al., 2011; Xenikou and Furnham, 2013). Transactional contingent reward puts effort into the development and maintenance of an organizational culture that encourages employees to work hard toward the accomplishment of their goals, allocates rewards primarily on the basis of individual effort expenditure and performance, and promotes a metaphor of the organization as a “marketplace” where one gets what she/he deserves (Bass and Avolio, 1993; Bass et al., 2003). In meta-analytic results transformational leadership has also been found to be a predictor of individual-level task performance, while transactional contingent reward predicted individual task performance beyond the effect of transformational leadership (Wang et al., 2011). Therefore, both transactional contingent reward and transformational leadership were hypothesized to be salient factors that contribute to employees' affective identification with the organization via a goal value orientation. The leader cultivates organizational values promoting individual goal setting and achievement orientation, which, in turn, are associated with positive emotional states at work and affective identification. On the grounds of the above theoretical analysis the following hypothesis was articulated (see Figure 1):

**Hypothesis 4**. Goal value orientation was expected to mediate the effect of transformational leadership and transactional contingent reward on affective identification.

### Overview of studies

Three studies were conducted in order to test for the hypothesized mediating effects of innovation and goal orientations in the link between leadership styles and organizational identification. In Study 1 a measurement of mediation design and a statistical mediation analysis with structural equation modeling (SEM) was employed to test for the research hypotheses. The remaining two studies (Study 2 and 3) together provided evidence for the central mediating role of individual perceptions of culture orientations by employing an experimental-causal-chain design (Spencer et al., 2005). Spencer et al. proposed that experimental designs should be used to examine mediation on the basis of the power of experiments in demonstrating causality; experiments are effective in establishing causal relations, and the specific case of establishing a mediator as the effect of an independent variable and the cause of a dependent is no different. Therefore, in Study 2 transformational leadership and transactional contingent reward were treated as independent variables and their causal effect on perceptions of value orientations was measured. Finally, in Study 3 innovation and goal value orientations were manipulated and their effect on cognitive and affective identification was measured. Together Studies 2 and 3 sought to test for the mediating role of organizational value perceptions in the relationship between leadership behavioral patterns and organizational identification utilizing an experimental-causal-chain design.

## Study 1

This first study sought to examine whether transformational leadership is directly, and indirectly via innovation and goal value orientations, related to cognitive and affective identification. Transactional contingent reward, on the other hand, was expected to be more strongly related to affective than cognitive identification with the employing organization, and the goal value orientation was hypothesized to statistically mediate the link between transactional contingent reward and the affective facet of organizational identification.

### Method

#### Participants

Participants were 172 full-time employees of diverse organizations in the public and the private sector, such as, schools, the army, and banks. There were 110 (64%) men and 56 (33%) women. Regarding their age, 65 (37%) were between 20 and 29 years old, 61 (36%) were between 30 and 39, 37 (22%) were between 40 and 49, and 9 (5%) were above 50. Concerning their education level, 34 (20%) were 6-year high school graduates, 92 (53%) were university graduates, and finally 46 (27%) had a postgraduate degree. In regard to their hierarchical position, 65 (38%) did not hold a management position and 106 (62%) were middle or upper level managerial personnel. Finally, regarding tenure with current employer, 102 (59%) were employed for more than 4 years, 24 (14%) were employed between 2 and 4 years, 16 (9%) between 1 and 2 years, and 29 (17%) were employed between 6 months and 1 year.

#### Measures

##### Culture value orientations

The second part of FOCUS Questionnaire (van Muijen et al., 1999) was used to measure employees' perceived organizational values. The subscale “innovation value orientation” includes flexibility, experimentation, as well as an external focus (8 items; sample items: risk taking, openness to criticism). The subscale “goal value orientation” concerns goal setting, achievement, performance indicators, and contingent reward (7 items; sample items: clear objectives, task oriented). All items are measured on a 6-point Likert scale (1 = not at all, 6 = to a very great extent). The Cronbach's alphas in this study were 0.72 for innovation and 0.76 for goal orientation.

##### Transformational/transactional contingent reward

The Multifactor Leadership Questionnaire (MLQ; Avolio et al., 1999) Form 5X was used to measure transformational leadership and transactional contingent reward. Participants were asked to describe their immediate supervisor's leadership on 24 items using a 5-point Likert scale (5 = frequently, if not always; 1 = not at all). Transformational leadership comprises three subscales, namely, charisma/inspirational (12 items), intellectual stimulation (4 items), and individual consideration (4 items), while the subscale of transactional contingent reward contains four items. The Cronbach's alphas in the current study were 0.84 for charisma, 0.80 for intellectual stimulation, 0.85 for individual consideration, and 0.85 for contingent reward. For the purposes of the current study, transformational leadership was treated as a higher order construct making no distinction between the three subscales. Finally, as individual employee perceptions of their immediate supervisor's leadership behavior were the focus of this study, transformational leadership and transactional contingent reward were treated as individual-level variables. Since the relevant literature reports moderate to high correlations between transformational leadership and transactional contingent reward possibly questioning the validity of their treatment as separate constructs, a Confirmatory Factor Analysis (CFA) for the two leadership styles was carried out. In the CFA transformational leadership was treated as a higher order construct with three subscales. The CFA results indicated problems concerning two items of the charisma/inspirational subscale, which were eliminated from any further analyses (descriptions of the conducted analyses can be provided by the author on request). CFA for transformational leadership as a higher-order factor with three subscales and transactional contingent reward demonstrated a good fit to the data, χ^2^_(196)_ = 263.53, *p* < 0.001; *CFI* = 0.95; *TLI* = 0.94; *SRMR* = 0.05, *RMSEA* = 0.04.

##### Cognitive/affective identification

Cognitive and affective organizational identification were measured by six items on a 7-point scale (1 = not at all, 7 = to a very great extent) developed by Ellemers et al. (1999) to tap the cognitive and emotional components of organizational identification. The cognitive component refers to a person's awareness of a particular social categorization, whereas the emotional component refers to a person's affective commitment to the group. The Cronbach's alphas in this study for cognitive and affective identification were 0.84 and 0.79, respectively.

#### Procedure

Participants were invited to take part in the study at their workplace by a research assistant, and were informed of the study's general purpose (i.e., examination of various important aspects of work life). Questionnaires were distributed to participants and were handed in to the research assistant, who returned to the workplace on three separate occasions to collect the filled questionnaires. A small number of organizational members who were asked to take part in the study refused to do so (approximately 11%). Participants did not have to provide their name on any part of the survey and the anonymity of their responses was ensured. The questionnaire took approximately 30 min to complete.

### Analytic strategy

SEM with ML estimation was used to test the research hypotheses, as implemented in Mplus (version 7.5). The formal significance tests of the indirect effects involved in the mediation models were carried out using the bias-corrected (BC) bootstrapped confidence interval (CI) method in order to address power problems introduced by non-normal sampling distributions of indirect effects (Hayes, 2013). This method involves the calculation of the indirect effect (a_1_b_1_) which is the product of the regression coefficient a_1_ that estimates the relation between the predictor and the outcome variable, and the regression coefficient b_1_ that estimates the relation between the mediator and the outcome. Following the calculation of the indirect effect, the bootstrapped CIs are calculated and in the case that the CIs do not include zero the null hypothesis is being rejected providing empirical support for the existence of a mediating effect.

### Results

Table 1 displays means, standard deviations, Cronbach's alphas, and correlations among study variables. The Cronbach's alphas of all the scales reached the acceptable criterion of 0.70 (Nunnally, 1978). Transformational leadership was significantly and positively correlated with innovation value orientation (*r* = 0.33) and goal value orientation (*r* = 0.43), as well as with cognitive (*r* = 0.35) and affective (*r* = 0.34) identification. On similar lines, transactional contingent reward showed positive correlations at a moderate level with the goal (*r* = 0.41) and innovation (*r* = 0.24) orientations. Moreover, all the organizational value orientations were significantly and moderately correlated with cognitive and affective identification. Finally, there was a significant positive correlation between transformational leadership and transactional contingent reward (*r* = 0.71), which indicates the complementary nature of the relationship between the two forms of leadership but posits the question of their relative validity.

**Table 1:** Means, Standard Deviations, Cronbach's alphas, and correlations among all relevant variables.

**Variables**	***M***	***SD***	**(1)**	**(2)**	**(3)**	**(4)**	**(5)**	**(6)**	**(7)**	**(8)**
Transformational leadership (1)	3.37	0.66	(0.92)							
Transactional contingent reward (2)	3.31	0.98	0.71^***^	(0.85)						
Innovation orientation (3)	3.78	0.85	0.33^***^	0.24^***^	(0.72)					
Goal orientation (4)	4.26	0.82	0.43^***^	0.41^**^	0.62^***^	(0.76)				
Cognitive identification (5)	3.74	1.43	0.35^***^	0.32^***^	0.36^***^	0.42^***^	(0.84)			
Affective identification (6)	4.95	1.49	0.34^***^	0.37^***^	0.49^***^	0.34^***^	0.37^***^	(0.79)		
Age^a^ (7)	2.94	0.92	−0.03	0.02	0.21^***^	0.15	0.16^*^	0.09		
Gender^b^ (8)	1.34	0.47	−0.04	−0.05	−0.31^***^	−0.18^*^	0.24^**^	0.02	−0.41^***^	
Organizational Tenure^c^ (9)	5.36	2.43	0.08	0.05	0.25^***^	0.16^*^	0.25^***^	0.19^*^	0.71^***^	−0.48^***^

*Internal consistency reliabilities are on the diagonal, in parentheses. N = 172*,

* *p ≤ 0.05*,

** *p ≤ 0.01*,

*** *p ≤ 0.001*.

a *Under 20 = 1; 20–29 = 2; 30–39 = 3; 40–49 = 4; 50–59 = 5; 60 and over = 6*.

b *Male = 1; Female = 2*.

c *Under 6 months = 1; 6 months to 1 year = 2; 1–2 years = 3; 2–4 years = 4; 4–6 years = 5; 6–10 = 6; 10–15 years = 7; Over 15 years = 8*.

Since this study employed a cross-sectional research design, it is possible that the findings may be subjected to multicollinearity. The variance inflation factor (VIF) was calculated for all the study variables in order to detect multicollinearity. The VFI statistic is the ratio of the total standardized variance over unique variance (tolerance), and it indicates that a variable is redundant when VFI exceeds the value of 10.0 (Kline, 2011). The generated VIFs for all the study variables ranged from 1.33 to 3.03 showing that multicollinearity was not present.

In addition, a Harman's single-factor test was conducted using CFA to address the issue of common-method variance (Podsakoff et al., 2003) in the current data. Harman's single-factor test examines whether a substantial amount of common-method variance is present by comparing a single-factor model with all items loading on it to a multi-factor model of the study variables. The single-factor model yield a bad fit to the data as shown by the following goodness-of-fit indices: $\chi_{\left(189\right)}^{2}$ = 895.85, *p* < 0.001; *CFI* = 0.50; *TLI* = 0.44; *SRMR* = 0.14, *RMSEA* = 0.15. More importantly, the six-factor model comprising transformational leadership, transactional contingent reward, innovation and goal orientation, and the two facets of organizational identification,^1^ i.e., cognitive and affective, fit the data well: $\chi_{\left(173\right)}^{2}$ = 264.49, *p* < 0.001; *CFI* = 0.94; *TLI* = 0.92; *SRMR* = 0.06, *RMSEA* = 0.05. These findings indicate that common-method variance should not be considered as a serious concern in the current study.

In order to test for the research hypotheses SEM with ML estimation was used, and a number of demographic variables, namely, age, gender and organizational tenure, that have consistently been shown to correlate with organizational identification (e.g., Mael and Ashforth, 1995) were used as control variables in the structural equation model. The mediation model showed an excellent fit to data: $\chi_{\left(26\right)}^{2}$ = 262.55, *p* < 0.001; *CFI* = 0.99; *TLI* = 0.96; *SRMR* = 0.03, *RMSEA* = 0.05. Moreover, all the parameter estimates associated with the study hypotheses were statistically significant. It may be noted that organizational tenure was positively related to cognitive identification, *b* = 0.11, *SE* = 0.05, *p* = 0.05, while gender was negatively related to innovation (*b* = −0.31, *SE* = 0.10, *p* = 0.01) and goal (*b* = −0.25, *SE* = 0.12, *p* = 0.05) orientation indicating that men compared to women perceived the culture of their employing organization as more innovative and goal-oriented.

Table 2 presents the results for hypotheses 1–4. Transformational leadership significantly predicted cognitive identification (c′_1_ = 0.49, *SE* = 0.19, *p* < 0.05) controlling for the effect of transactional contingent reward. On the other hand, there was no direct effect of transformational leadership on affective identification (c′_2_ = −0.16, *SE* = 0.19, *p* = ns) when controlling for transactional contingent reward. Therefore, hypothesis 1 was partially supported. Transformational leadership qualities such as, intellectual stimulation, cognitive flexibility, individualized support, and ethical standards/integrity were shown to be associated with employees' tendency to think of themselves in terms of their organizational membership and focus on perceived similarities between the self and other group members rather than differences. At this point one has to note that transactional contingent reward was not a predictor of cognitive identification, c′_3_ = 0.05, *SE* = 0.17, *p* = ns, when controlling for the effect of transformational leadership.

**Table 2:** Mediation structural equation model results.

**Predictor variables**	**Outcome variable: innovation orientation**			**Outcome variable: goal orientation**		
	***b***	***SE***	***P***	***b***	***SE***	***P***
Age	0.02	0.11	0.837	0.03	0.10	0.792
Gender	−0.31	0.10	0.003	−0.25	0.12	0.034
Organizational tenure	0.03	0.04	0.390	−0.01	0.04	0.953
Transformational leadership	0.42	0.11	0.000	0.18	0.17	0.279
Transactional contingent reward	0.01	0.08	0.883	0.26	0.11	0.013
**Predictor variables**	**Outcome variable: cognitive identification**			**Outcome variable: affective Identification**		
	***b***	***SE***	***P***	***b***	***SE***	***P***
Age	−0.05	0.17	0.785	−0.02	0.17	0.905
Gender	0.07	0.22	0.767	0.34	0.20	0.090
Organizational tenure	0.11	0.05	0.051	0.12	0.07	0.072
Transformational leadership	0.49	0.19	0.035	−0.16	0.19	0.416
Transactional contingent reward	0.05	0.17	0.781	0.44	0.16	0.006
Innovation orientation	0.45	0.13	0.001	0.18	0.16	0.261
Goal orientation				0.62	0.15	0.001

Hypothesis 2 suggested that transactional leadership qualities are positively related to affective identification after controlling for the effect of transformational leadership style. Indeed transactional contingent reward was positively related to affective identification while controlling for the effect of transformational leadership (c′_4_ = 0.44, *SE* = 0.16, *p* < 0.01) providing support for Hypothesis 2.

Moreover, we investigated whether organizational culture orientations statistically mediated the links between leadership styles and organizational identification using a bootstrapping procedure with 5,000 resamples (Hayes, 2013). Results showed that there was an indirect effect of transformational leadership on cognitive identification through innovation orientation, a_1_b_1_ = 0.19, CI 95% [0.03, 0.32], whereas the indirect effect of transformational leadership on affective identification via innovation was not significant, a_1_b_2_ = 0.08, CI 95% [−0.07, 0.22], partially supporting Hypothesis 3. There was also an indirect effect of transactional contingent reward on affective identification via goal orientation, a_3_b_3_ = 0.16, CI 95% [0.01, 0.32], while the indirect effect of transformational leadership on affective identification via goal orientation was not significant, a_2_b_3_ = 0.11, CI 95% [−0.10, 0.32], partially supporting hypothesis 4.

The results of the mediation analysis offer partial support to hypotheses 3 and 4. As far as hypothesis 3 is concerned, the results presented in Table 2 show that innovation orientation was found to mediate the relation between transformational leadership and cognitive identification. With regard to hypothesis 4, goal value orientation was found to be a mediator of the relationship between transactional contingent reward and affective identification.

A review of the sizes of direct and indirect effects show that transformational leadership was consistently related to cognitive identification, while transactional contingent reward was directly and indirectly related to affective identification. The direct effect (c′) is the effect of the predictor on the outcome controlling for the mediator and the indirect effect (a^*^b) is the effect of the predictor on the outcome variable via the mediator. The total effect of each of the leadership style variables is the sum of the indirect effect of the predictor on the outcome variable via the mediator and the direct effect. For transformational leadership, there was a significant direct effect on cognitive identification (c′_1_ = 0.49, *p* < 0.05) and a significant indirect effect via innovation (a_1_b_1_ = 0.19; total effect = 0.68). For transactional contingent reward, there was significant direct effect on affective identification (c′_4_ = 0.44, *p* < 0.01) and a significant indirect effect via goal (a_3_b_3_ = 0.16; total effect = 0.60).

### Discussion

The findings of Study 1 show that transformational leadership had a positive direct effect, and an indirect effect via innovation culture orientation, on cognitive identification. Transactional contingent reward, on the other hand, was more strongly related to affective rather than cognitive identification, and the goal value orientation mediated the link between transactional contingent reward and affective identification. Study 2 aimed to provide a first step in a more elaborate test of the research hypotheses by implementing a causal mediation design.

## Study 2

Study 2 was an experiment in which participants were asked to read an interview with the general director of a construction company published in a local newspaper, and imagine they were employed by this company having the interviewee as their supervisor. Participants were also told that in the interview the director was describing the way he understands his role as a manager and how he should be interacting with subordinates. Subsequently, participants were asked to rate the organizational culture of the construction company with regard to innovation and goal value orientations. It was expected that participants in the transformational leadership condition to rate the culture of the organization as more innovation-oriented compared to participants in the transactional contingent reward condition (hypothesis 1). Moreover, it was expected that participants in the transactional contingent reward condition to perceive the organizational culture as more goal-oriented than innovation-oriented (hypothesis 2).

### Method

#### Participants and procedure

Participants were 44 military cadets who took part in the study for course credit. There were 42 men and 2 women. Their age ranged from 18 to 21 years old (*M* = 20.27, *SD* = 0.50). Participants were informed that the main objective of this study was to explore the linkages between different leadership styles and subordinates' work attitudes and motivation. Following reading the interview extracts, they were asked to rate the organizational culture of the construction company on the innovation and goal value orientations. Participants did not have to provide their names, and were informed of their right to withdraw from the study at any stage of the process.

#### Manipulation of leadership styles

The transformational and transactional contingent reward interview extracts were based on leadership scenarios developed by Hamstra et al. (2014).

The transformational leadership scenario reads as follows:

For many years now, I have worked in a supervisor position. My success as a leader stems from my dedication to the future of this organization and I try my best to communicate my vision to the people who work here. I often express the opinion that I find it important that people do their best and set high goals. However, this does not mean all I care about is productivity. For me it's really about employees developing themselves and being recognized as individuals. I also think it is important to do things differently than this organization has done in the past. I like to take some risks in showing how things could be improved. I rely on my employees to find new ways of working. Therefore, I value their creative and intellectual input, and stimulate them to provide their own ideas. I expect my employees to view their work as more than just a job: to feel that they are part of something special, something great and important.

The transactional leadership reward scenario reads as follows:

For many years now, I have worked in a supervisor position. My success as a leader comes from the fact that I provide employees with clarity. I make clear to people what they are expected to do and which goals to achieve in order to obtain desired rewards. My employees know that the relationship I have with them is based on reciprocity. I make agreements with people about what I expect from them and what they should achieve in their work and I expect people to live up to those agreements. In order to make sure that employees live up to agreed-upon standards, I generally keep a close eye out to see that everything goes well. I think it is important to maintain the status quo in this company and to ensure stability. Therefore, I draw attention to what needs to be done as well as the standards of evaluating the quality work, and given that these standards are met I offer the analogous rewards.

The manipulation of leadership patterns was pretested in 44 military cadets (95% men, *M*_age_ = 20.61, *SD*_age_ = 0.58), who were asked to read the interview extracts of transformational and transactional contingent reward leadership and then rate the leadership styles using the MLQ (Avolio et al., 1999; transformational leadership *M* = 3.69, *SD* = 0.41; *a* = 0.76; transactional leadership *M* = 3.77, *SD* = 0.71; *a* = 0.71). The results showed that the leader in the transformational leadership interview extract was rated as more transformational than the leader in the transactional contingent reward extract, *t*_(40)_ = 2.44, *p* < 0.01 (*M* = 3.83 vs. *M* = 3.54), and more transformational than transactional, *t*_(20)_ = 3.49, *p* < 0.01 (*M* = 3.85 vs. *M* = 3.37). In addition, the leader in the transactional contingent reward interview extract was rated as more transactional than the leader in the transformational interview extract, *t*_(41)_ = −4.31, *p* < 0.001 (*M* = 4.15 vs. *M* = 3.37), and more transactional than transformational, *t*_(19)_ = −4.23, *p* < 0.001 (*M* = 4.21 vs. *M* = 3.54).

#### Measures

Innovation and goal value orientations were assessed with the same measure as in Study 1. Innovation (*M* = 3.67, *SD* = 0.96; *a* = 0.83) and goal (*M* = 4.74, *SD* = 0.61; *a* = 0.73) value orientations were reliable scales and their correlation was *r*_(39)_ = −0.11, *p* = ns.

### Results

Simple comparisons showed that participants in the transformational leadership condition rated the culture of the company to be more innovation-oriented compared to the transactional contingent reward condition supporting hypothesis 1, *t*_(39)_ = 3.56, *p* < 0.001 (*M* = 4.15 vs. *M* = 3.21; effect size^2^ = 4.15–3.21/0.84 = 1.12). Additionally, in the transformational leadership condition participants perceived the culture to be more goal-oriented than innovative, *t*_(18)_ = 2.42, *p* < 0.05 (*M* = 4.63 vs. *M* = 4.16; effect size = 4.63–4.16/0.67 = 0.70). Participants in the transactional contingent reward condition rated the culture as more goal-oriented than innovative, *t*_(19)_ = 5.76, *p* < 0.001 (*M* = 4.78 vs. *M* = 3.17; effect size = 4.78–3.17/0.81 = 1.99) offering support to hypothesis 2. Finally, there was no difference in goal-orientation ratings between the transactional contingent reward and the transformational leadership conditions, *t*_(39)_ = 0.72, *p* = ns (*M* = 4.81 vs. *M* = 4.67; effect size = 4.81–4.67/0.61 = 0.23).

### Discussion

This experimental study provided evidence for the part of the hypothesized causal chain which postulates that transformational leadership behaviors lead employees to perceive the culture of the organization as more innovative than transactional contingent reward. Moreover, the proposition that transactional contingent reward leads employees to perceive the culture as more goal-oriented than innovative was also supported.

## Study 3

Study 3 aimed to provide evidence for the second part of the causal chain by manipulating innovation and goal organizational value orientations and examining their effect on cognitive and affective identification with the organization. We expected participants in the innovation value condition to show higher levels of cognitive identification compared to goal value condition (hypothesis 1), whereas participants in the goal orientation to identify with the employing organization more in affective rather than cognitive terms (hypothesis 2). Importantly, in order to ensure that measurement (Study 1) and manipulation (Study 3) of organizational value orientations represent the same construct, the measurement and manipulation of value orientations in both studies were based on the same items taken from FOCUS Questionnaire (van Muijen et al., 1999).

### Method

#### Participants and procedure

Participants were 40 military cadets (38 men and 2 women) taking part in the experiment for course credit. Their age ranged from 19 to 21 years old (*M* = 20.07, *SD* = 0.35). They were informed that the study investigated perceptions of organizational climates and cultures that emerge in different work environments, and their relation to employees' work attitudes. Participants were presented with the innovation or goal organizational culture of a construction company and were asked to vividly imagine that they were employed by this company. Finally, they completed measures of cognitive and affective identification with the organization.

#### Manipulations and measures

##### Culture value orientations

The manipulation of organizational value orientations was conducted using the list of items from the FOCUS Questionnaire (van Muijen et al., 1999) measuring innovation (8 items) and goal (7 items) value orientations that were also used in Studies 1 and 2. In the experimental-causal-chain design it is important that the manipulation of the process is the same variable as the measurement of the process, therefore it is imperative that the same items of FOCUS Questionnaire are used in Studies 3 and 2. As mentioned above, participants were asked to vividly imagine that they were employed by a company in which the work climate/culture encourages employees to engage in the behaviors describing either an innovation (e.g., risk taking) or goal (e.g., clear objectives) value orientation.

##### Organizational identification

Organizational identification was assessed with the same measure used in Study 1, slightly adapted to the experimental context (cognitive identification: *M* = 4.11, *SD* = 1.04, *a* = 0.72; affective identification: *M* = 5.58, *SD* = 1.20, *a* = 0.87).

### Results

Simple comparisons provided evidence that participants in the innovation orientation condition showed higher cognitive identification with the organization in comparison to the goal orientation condition providing support to hypothesis 1, *t*_(37)_ = 3.31, *p* < 0.01 (*M* = 4.61 vs. *M* = 3.63; effect size = 4.61–3.63/0.88 = 1.11), and identified with their organization more in affective rather than cognitive terms, *t*_(18)_ = 6.14, *p* < 0.001 (*M* = 6.16 vs. *M* = 4.61; effect size = 6.16–4.61/0.64 = 2.42). In the goal orientation condition participants identified with the employing organization more in affective rather than cognitive terms supporting hypothesis 2, *t*_(19)_ = 5.69, *p* < 0.001 (*M* = 4.97 vs. *M* = 3.63; effect size = 4.97–3.63/1.14 = 1.18), and showed lower affective identification compared to the innovation orientation condition, *t*_(24.85)_ = 3.73, *p* = 0.001 (*M* = 6.20 vs. *M* = 4.97; effect size = 6.20–4.97/1.04 = 1.18).

### Discussion

Study 3 provided empirical support for the second part of our causal chain by experimentally manipulating the mediator, namely, innovation and goal value orientations, and measuring the outcome variable. Manipulating innovation and goal value orientations led participants to report different forms of psychological relatedness to the employing organization, such as an innovation orientation to enhance perceptions of similarity among organizational members (cognitive identification) in comparison to a goal orientation, whereas a goal orientation to boost affective, rather than cognitive identification.

## General discussion

In this research perceptions of organizational culture and transformational/transactional leadership were examined as antecedent factors of the psychological bond between individuals and organizations. Building on an important theoretical account of organizational identification that places emphasis on the self-engaging processes of transformational/charismatic leadership (Shamir et al., 1993; Lord et al., 1999) the main aim was to investigate how transformational/transactional leadership styles and culture orientations influence individual employees' cognitive/affective identification with the employing organization. The findings provide support for the relative validity of transformational leadership and transactional contingent reward as predictors of employees' bonding with the organization they work for. Transformational leadership behaviors were found to predict cognitive identification controlling for the effect of transactional contingent reward, whereas transactional contingent reward was related to affective identification when the effect of transformational leadership was accounted for. The results of the two experimental studies further support these findings by demonstrating in Study 2 that transformational leadership led to the perception of culture as more innovation-oriented in comparison to transactional contingent reward, while transactional contingent reward led participants to perceive the culture as more goal-oriented than innovative. In addition, the second part of the causal-mediation-analysis carried out in Study 3 demonstrated that innovation value orientation in comparison to goal orientation led to higher cognitive identification, whereas goal orientation increased affective, rather than cognitive, identification.

The finding that transactional contingent reward was a significant predictor of affective identification controlling for the effect of transformational leadership indicates that transformational leadership is not a substitute for transactional contingent reward. Transactional leadership behaviors were shown to further enhance the emotional bonding between individual employees and their organization beyond the positive effect of transformational leadership behaviors. These findings offer support to the proposition of Wang et al. (2011) that transformational leadership and transactional contingent reward are complementary forms of leadership that work through different mechanisms to facilitate the development of positive employee attitudes and to enhance effective performance. Previous research has suggested that transformational leadership behaviors involve intellectual stimulation and creative behavior, such as suggesting novel ways to solve problems and different angles to address challenges (Shin and Zhou, 2007; Gong et al., 2009; Henker et al., 2015), which, in turn, have a positive impact on organizational cognitive identification. The cognitive flexibility associated with transformational leaders exhibiting intellectual stimulation in re-framing the situation and looking at old problems from new angles is conducive to cognitive identification. Individual employees who are supervised by transformational leaders tend to perceive themselves as more similar to other members of the organization on the grounds of cognitive flexibility and inclusiveness evoked by the leader promoting and encouraging creative and innovative behaviors.

Moreover, transactional contingent reward was found to be positively related to affective identification beyond any effect of transformational leadership. Therefore, when task and role responsibilities are clarified by leaders, and support, as well as rewards, are provided to followers for their efforts and accomplishments, the emotional bonding between individual employees and the organization is strengthened. This finding appears to be in line with the results of Schriesheim et al. (2006) and Vecchio et al. (2008) that transactional contingent reward was significantly related to individual follower performance after controlling for transformational leadership. On similar lines, Wang et al. (2011) provided evidence that transactional contingent reward predicted individual task performance beyond the effect of transformational leadership.

A possible interpretation of this finding is that transactional contingent reward evokes positive activation by helping employees to set and achieve various work goals, such as successful task completion, promotion, and a pay rise. Organizational members develop an emotional attachment with their organization possibly via the experience of positive affect, such as feeling energetic, enthusiastic, and excited. In accordance with the social exchange perspective in the study of organizations, transactional contingent reward explained variance in affective identification beyond any variance accounted by transformational leadership behaviors. Social and economic exchanges provided by transactional leadership behaviors were associated with employees' emotional attachment to the organization. These findings are also in line with those reported by Tyler and Blader (2003) and Blader and Tyler (2009) showing that the level of resources that employees receive from the organization shaped organizational identity, which, in turn, was related to extra-role behavior integrating the social identity and the social exchange perspectives. Therefore, it seems that the quality of the exchange relationship with the organization as enacted by its leaders has a stronger impact on affective, rather than cognitive, identification. In other words, members' feelings of affective identification with the employing organization are shaped by the evaluation of the resources and rewards that the organization offers to its employees through the transactional style of leadership possibly associated with perceptions of justice and equality.

As leaders may partly shape organizational cultures by providing direction and coherence, employees' identification with the organization was expected to be affected by the development, cultivation, and change of value priorities by organizational leaders. To test for the mediating effect of culture orientations in the relation between transformational leadership and organizational identification, this research utilized a comprehensive framework of the organizational culture dimensions that has received extended empirical support (Quinn, 1988; Cameron and Quinn, 1999; Vandenberghe and Peiro, 1999; van Muijen et al., 1999; Meyer et al., 2010; Hartnell et al., 2011). The findings showed that innovation value orientation mediated the relationship between transformational leadership and cognitive identification. Therefore, when employees perceive their immediate supervisor to promote innovation and creativity among organizational members the attributes which are cognitively selected for comparison of the self to the organization (or its members) are biased toward producing assimilation rather than contrast effects. Since assimilation rather than contrast is the default process in social judgments (Mussweiler, 2003), an innovation value orientation may possibly reinforce this default social process in organizational settings leading to strong cognitive ties between the self and the organization.

The findings also supported the hypothesized mediating effect of goal value orientation in the relation between transactional contingent reward and affective identification. A goal culture orientation involves value preferences for individual goal setting, performance measurement, and contingent reward (Quinn, 1988; van Muijen et al., 1999; Hartnell et al., 2016), which tend to reflect an approach motivational state. Striving for the accomplishment of goals has been demonstrated to lead to the experience of positive affect which, at high levels of intensity, reflects the extent to which a person feels active, alert, and enthusiastic, while at a low level of intensity reflects sadness and depression. The findings of this study showed that transactional contingent reward was directly, as well as indirectly, related to higher levels of employees' emotional attachment to the organization via the mechanism of goal value orientation. Specifically, the transactional leader by clarifying role expectations, offering constructive feedback, and rewarding individual accomplishments shapes the beliefs employees hold regarding the extent to which the organization adopts a goal value orientation, which, in turn, influences their desire to be affiliated with the organization.

With regard to strengths of this research, implementing both measurement and causal mediation designs as suggested by Spencer et al. (2005) allowed for making inferences about the existence of causal relationships. The conceptual elaboration and empirical support of the proposed research hypotheses on the grounds of correlational data did offer higher ecological validity of the findings, and certainly facilitated the unraveling of causal relationships. Nevertheless, to make inferences about causal relations it is necessary to utilize research designs characterized by internal validity, that is, experimental designs or longitudinal research. The higher internal validity of experimental studies, even compared to longitudinal designs, is attributed to their power to control for confounding variables via the random allocation of participants to experimental conditions. Therefore, the combination of measurement and causal mediation designs added extra validity to the findings of this research.

A possible limitation concerns the sample of Study 1, which, even though, was heterogeneous with employees from different industry sectors, organizations, and occupations, offers limited confidence in the generalizability of the reported findings. Similarly, in experimental studies 2 and 3 the samples comprised predominantly male participants while female subjects were under-represented. Therefore, the replication of the findings, with different and more diverse samples, is a goal of future research in this field. In addition, one should make a note on the limitation of self-report measures used for data collection, but it is employees' subjective perceptions measured by self-report techniques that are arguably the most important predictors of individual employee attitudes. Despite the limitations noted above, this research represents a first step toward unraveling both direct and indirect effects of transformational leadership and transactional contingent reward on cognitive and affective identification, and the mediating role of perceptions of organizational culture.

The practical implications of these findings involve the different strategies organizations can develop to strengthen their ties with their employees, which, in turn, may have a positive impact on a series of in-role and extra-role behaviors. The implementation of fair transactional exchanges as agreed upon by transactional leaders and their followers is conducive to the emotional attachment of individual employees to their organizations. A culture orientation promoting goal setting, achievement, and contingent reward is a path through which transactional leaders help employees develop positive feelings toward the employing organization. Moreover, transformational leadership, as a complementary leadership style interlinked with transactional contingent reward, is a key element in nurturing a culture of innovation and change, which leads employees to perceive themselves as more similar to the organization, as well as to other organizational members, and to work toward the organization's best interests.

## Ethics statement

All subjects participating in this research project gave their written informed consent, and were reminded of their right to withdraw from the study at any time during the process.

## Author contributions

The author confirms being the sole contributor of this work and approved it for publication.

### Conflict of interest statement

The author declares that the research was conducted in the absence of any commercial or financial relationships that could be construed as a potential conflict of interest. The reviewers and handling Editor declared their shared affiliation.

## Footnotes

^1^The construct structure of organizational identification was checked by comparing the six-factor model incorporating the two dimensions of organizational identification that is, cognitive and affective, to an alternative five-factor model representing organizational identification as uni-dimensional. CFA results showed that the five-factor model did not fit the data well: $\chi_{\left(176\right)}^{2}$ = 347.41, *p* < 0.001; *CFI* = 0.88; *TLI* = 0.86; *SRMR* = 0.07, *RMSEA* = 0.08.

^2^Effect size (ES) for each simple comparison has been calculated using the following formula:

$$\begin{array}{l} \text{ES}=\frac{\text{Mean Grou}{\text{p}}_{1}\;-\;Mean\;Group_{2}}{\text{SD pooled}}. \end{array}$$
